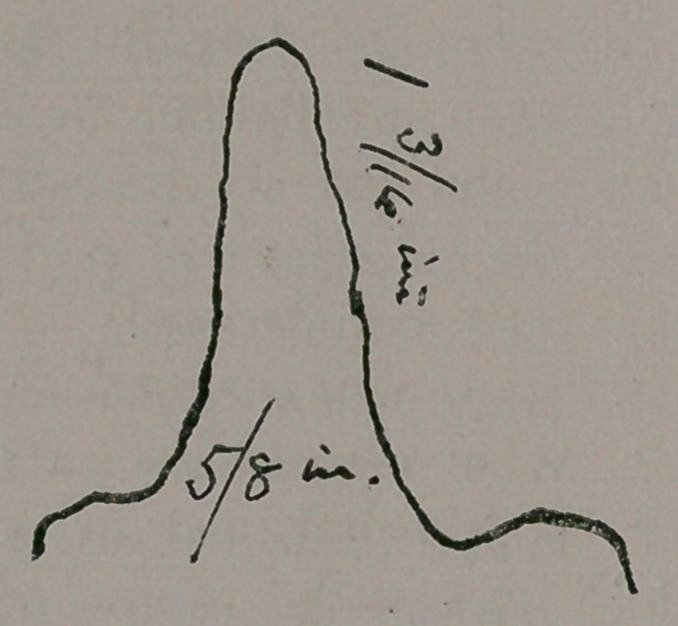# Staphylorraphy*Read before The Homœopathic Medical Society of the County of New York, November 9th, 1893.

**Published:** 1894-01

**Authors:** Edmund Carleton

**Affiliations:** New York


### STAPHYLORRAPHY:* A CASE FROM PRACTICE.

EDMUND CARLETON, M. D., NEW YORK.

Mr. McC. came from Pennsylvania for operation and was brought to me by Dr. E. C. J. Tappen. He was eighteen years old. The accompanying outline sketch shows the gap which had to be filled—one inch and three-sixteenths long on the shortest side; greatest breadth five-eighths of an inch.

Of course his speech was very poor; and while hopes were held out that closure could be effected in part, he was cautioned not to expect much improvement in talking, as it is almost impossible at eighteen years of age to overcome the bad habits of articulation acquired in childhood.

For a number of days before the operation, he faithfully tickled his throat with a feather and rubbed it with a swab, to make it tolerant of manipulation.

At the appointed time, April 14th, 1893, the patient sat in an ordinary chair facing the window, which gave me an opportunity to sit facing him with my back to the window and to have plenty of light shine directly into the throat. Besides Dr. Tappen and Dr. W. L. Allen (an old school friend), our colleague, Dr. Fralick, gave his valuable assistance.

The Whitehead gag having been put between the jaws and well opened, the parts were brushed with cocaine. The right

* Read before The Homœopathic Medical Society of the County of New York, November 9th, 1893.

flap (patient's right) was seized at its extremity with a tenaculum forceps and held tense, and then pared with a slender blade, beginning with transfixion above and ending with a downward cut. This, at my hands, gives a better result than the ordinary upward cut, although the necessity of working rapidly is not overlooked, to avoid obscuration of the path of incision from hemorrhage. Then the sponge mop. Then the other side trimmed in like manner, so that the entire portion removed was one slender piece. Then the gag was removed and patient requested to gargle until hemorrhage stopped.

N. B.—All sponging and gargling during this operation was with Croton water from the warm faucet, medicated with tincture of Calendula, a tablespoonful of the latter to a quart of water.

In a few minutes the patient was ready for the gag once more. Four silver sutures were then introduced, from patient's left to right, by means of a tubular needle, curved an inch from point backward, and then made rectangular to the rest of itself and the handle. After the last stitch had been taken, which was the lowest, the first (upper) stitch was found to be broken, so great had been the tension at that point, and had to be renewed.

The next important step was to divide the levator and tensor palati muscles. This was done by inserting a tenotome just inside the hamular process, and carrying the blade upward with a sweeping movement until both muscles were severed. The careful observer of the outline sketch has noticed that the left side was larger than the right (patient's left, right side upon paper). All along, I had cherished the idea that somehow this could be turned to advantage. And so it was. By dividing the muscles upon the right side only, tension was removed, and at the same time the parts were brought into such relation that a respectable little uvula appeared.

After another careful gargling, final instructions were given not to use the muscles of the throat, either for speech or swallowing, for eight days. Liquid food was allowed to run down into the stomach without effort.

Six days later, a small point was found ununited. This was touched with nitrate of silver, five days in succession, until firm union was assured. Then *Natrum-muriaticum* in potency, was given to antidote *Argentum-nitricum.* The stitches were removed on the eighth day.

As soon as he was permitted to use his vocal organs, this young man went to work intelligently and systematically, under instruction, to master the difficulties that lay before him. Already he speaks rather plainly, and does not skip the gutturals when he comes to them. At this rate, he will talk naturally at the end of a year. I ascribe our success in a large measure to him. The preparation of the case, careful attention to details, and use of *Calendula* are of first importance in my estimation.

—

LATER.—Under date of November 5th, 1893, he writes: "I can speak much plainer than I have done, and am improving all the time."

## Figures and Tables

**Figure f1:**